# Extracorporeal Photopheresis in Dermatological Diseases

**DOI:** 10.3390/ijms25053011

**Published:** 2024-03-05

**Authors:** Hanna Terhaar, Mohammad Saleem, Nabiha Yusuf

**Affiliations:** 1 Heersink School of Medicine, University of Alabama at Birmingham, Birmingham, AL 35294, USA; hterhaar@uab.edu; 2 Department of Dermatology, University of Alabama at Birmingham, Birmingham, AL 35294, USA; msaleem@uabmc.edu

**Keywords:** extracorporeal photopheresis, extracorporeal photochemotherapy, autoimmune skin diseases, immunotherapy, dermatologic diseases

## Abstract

Extracorporeal photopheresis (ECP) is an apheresis procedure that is conventionally used as a first-line treatment for cutaneous and leukemic subtypes of T-cell lymphoma, such as Sezary’s syndrome and mycosis fungoides. Over the past three decades, its immunotherapeutic properties have been tested on a variety of autoimmune conditions, including many dermatologic diseases. There is ample evidence of ECP’s ability to modify leukocytes and alter cytokine production for certain dermatologic diseases that have been refractory to first-line treatments, such as atopic dermatitis. However, the evidence on the efficacy of ECP for the treatment of these dermatologic diseases is unclear and/or lacks sufficient evidence. The purpose of this paper is to review the literature on the utilization and clinical efficacy of ECP in the treatment of several [autoimmune] dermatologic diseases and discuss its applications, guidelines, recommendations, and future implementation for dermatologic diseases.

## 1. Introduction

### 1.1. Photopheresis

Extracorporeal photopheresis is a nonsurgical procedure that was first introduced in 1988 as a way to treat cutaneous T-cell lymphoma (CTCL) [1]. However, over the past three decades, ECP has been utilized as a therapeutical treatment for a variety of diseases, along with hematopoietic stem cell transplantation and graft vs. host disease. During an ECP procedure, plasma is extracted via a cubital vein or a permanent catheter and flows into the photopheresis device. The photopheresis device utilizes centrifugation to separate the leukocytes or “buffy coat” from the rest of the plasma. The rest of the plasma is returned to the patient while the separated leukocytes are then treated with 8-methoxypsoralen (8-MOP), also called psoralen, a compound used to increase the amount of UV light that is absorbed in the patient’s leukocytes, commonly found in lemons and figs [2]. Once the white blood cells are chemically treated, they are then exposed to ultraviolet light (UVA), ranging from 329 to 400 nm, which activates the 8-MOP, which forms cross-links in the DNA, inducing cell injury and apoptosis [3]. These treated leukocytes are then reintroduced back into the patient, where the patient’s immune system targets the damaged leukocytes for apoptosis [4].

### 1.2. ECP Mechanism of Action

There is some debate as to whether or not the amount of apoptotic material reintroduced to the patient’s blood will outcompete the patient’s immune system’s ability to clear the apoptotic debris from their system. The massive amount of uncleared apoptotic debris can then go on to wreak havoc on the immune system by inducing pathways such as cell necrosis, auto-antibodies to the uncleared apoptotic material, and an increase in other inflammatory factors, which could potentially result in autoimmune disorders such as systemic lupus erythematosus (SLE). However, scientists such as Renzo et al. have found that rarely do patients with a massive amount of apoptotic debris in a patient’s system go on to develop clinical autoimmune diseases [5,6]. Additionally, there are no reports that show that ECP procedures can lead to the onset of autoimmune diseases. 

## 2. ECP in Dermatologic Diseases

Although extracorporeal photopheresis is widely known to be approved as a first-line treatment for the CTCL variants, such as Sezary syndrome and mycosis fungoides, the American Society For Apheresis guidelines (ASFA) has recognized ECP as a treatment for several dermatologic diseases, with specificity for dermatologic diseases with autoimmune origin [7]. Table 1 presents the European Dermatology Forum (EDF) and the American Society for Apheresis (ASFA) guidelines for the treatment of dermatological diseases that have explored the use of ECP as a potential therapy [7,8]. The stage and grading of the quality of evidence for the ASFA recommendations are depicted in Supplementary Tables S1 and S2.

### 2.1. Atopic Dermatitis

Atopic dermatitis (AD) is a chronic eczematous disease that affects an estimated 16.5 million U.S. adults, with about 40% diagnosed with a moderate to severe course of the disease [9]. Patients usually present with erythematous papules localized to the face, trunk, extremities, and flexor surfaces. Patients can also present with dry, scaly patches on their extremities as well as lichenization, which presents as thick and leathery patches of skin due to chronic itching. Although the mechanism of action behind atopic dermatitis is not completely understood, it is universally understood as a multifactorial disease, with genetic and environmental factors playing a role in the onset and persistence of this disease. A possible genetic cause that might play a role in the onset of AD in a subset of patients includes a genetic defect in the FLG gene, which is responsible for the production of filaggrin protein, which normally serves as a part of the skin protection barrier that maintains hydration to the skin [10]. This causes an impairment in the permeability of the epidermis, allowing antigens to diffuse into the lower layers of the epidermis and dermis, activating Th2 cells, which produce IL-4 and IL-13, ultimately leading to an inflammatory response [10]. Many first-line treatments, such as calcineurin inhibitors and corticosteroids, have been utilized in order to treat this debilitating condition; however, several moderate to severe AD patients are recalcitrant to these therapies, especially if AD is chronic and widespread.

ECP, for the treatment of AD, has been explored as early as 1994 [11]. A summary of each of the ECP treatments for AD is depicted in Table 2.

Prinz et al. observed that patients showed a noticeable decrease in skin lesions and an improvement in pruritic symptoms and erythema [11]. Additionally, the laboratory results showed a decrease in IgE in all three patients with stable IgG, IgA, and IgM levels, suggesting that ECP may interfere with the pathogenic mechanisms behind AD [11]. Over the next three decades, many prospective, retrospective, and case reports that followed have all validated the previous finding that ECP has been proven effective in the treatment of severe AD (*n* = 167). Many of the studies followed the EDF recommendation for initial treatment scheduling and administered ECP in 2-week intervals for 12 weeks and decreased the ECP treatments thereafter. Prospective trials that proved ECP efficacious include Wolf et al., who conducted ECP treatments over a 20-week period on 10 AD patients that had a SCORAD of >45 and were refractory to first-line therapies. The results show that there was a significant decrease in the mean of the SCORAD of all patients by week 20 [18]. Additionally, a recent study conducted by Gambichler et al. found measured blood parameters at 3-month intervals up to 1 year of treatment in 60 AD patients treated with ECP. Leukocytes and lymphocytes were found to remain decreased after one year of ECP treatment [23]. Additionally, there was a significant decrease in eosinophils and eosinophil cationic protein levels, along with IgE and lactate dehydrogenase levels [23].

Accumulation of the positive findings has led to the EDF labeling ECP as a second-line treatment for refractory and severe AD. The EDF states that the initial treatment dosage is recommended to be one cycle every 2 weeks for 12 weeks. Maintenance schedules should be tailored to the patient; however, the goal is to taper the ECP treatment to one cycle every 6–12 weeks [8]. Clinical assessment includes a biweekly SCORAD assessment for the first 12 weeks and then at monthly or longer intervals. In contrast, AFSA has acknowledged that ECP could be a beneficial treatment based on the previously published high-quality literature; however, there is not enough evidence for AFSA to confidently label ECP as a first- or second-line treatment for AD. Therefore, more studies on the efficacy of ECP on refractory AD patients should be conducted in order to verify the observations of previously published literature.

### 2.2. Cutaneous Lupus Erythematosus

Cutaneous lupus erythematosus (CLE) is a subtype of lupus erythematosus (LE) that solely affects the skin. This autoimmune disease has ten subtypes, including acute cutaneous lupus, subacute cutaneous lupus, and discoid lupus [24]. Acute cutaneous lupus is the typical “butterfly rash” that is seen in systemic lupus erythematosus (SLE), which usually involves autoimmune destruction of other organs. Subacute cutaneous lupus is solely limited to the skin and is characterized by red/pink polycyclic annular patches or plaques seen in sun-exposed areas. Discoid lupus is normally limited to the face and is described as red macules/plaques that evolve into atrophy, scarring, and pigment changes and rarely have mucosal involvement [24]. Evidence of the use of ECP for the treatment of LE is depicted in Table 3.

A case series conducted by Knobler et al. utilized 10 patients diagnosed with SLE and treated the patients with two ECP treatments every 4 weeks for 6 months, followed by one treatment every 8 weeks for 6 months. Only 8 out of the 10 patients could complete the study, as one dropped out for personal reasons, and another patient passed away during the study [25]. The results of the study showed that at 4–6 months, seven out of the eight patients showed significant improvement in their skin manifestations, such as discoid rash, alopecia, and photosensitivity, with an improvement in their arthralgias. These patients were able to decrease their dose of immunosuppressants and steroids. It was also noted that there were no notable changes in the serology measurements during or after this trial [25]. The remaining evidence of ECP on LE treatment is limited to case reports with chronic CLE patients (*n* = 3), subacute cutaneous lupus (*n* = 4), discoid lupus erythematosus (*n* = 2), and SLE accompanied with urticarial vasculitis (*n* = 1) [27,28,29,30,31,32]. All the case studies reported positive effects on ECP, including complete remission in several patients, with partial remission in others, regardless of photosensitivity.

Despite the evidence that shows that cutaneous lupus erythematosus can be treated successfully with ECP, the ASFA has not published guidelines on the use of ECP for cutaneous lupus erythematosus, most likely due to the lack of external validity. However, the EDF guidelines state that the preliminary results prove to be an effective and safe option for lupus erythematosus.

### 2.3. Dermatomyositis

Dermatomyositis (DM) is a rare idiopathic disease that affects about 200,000 individuals in the United States [33]. DM is characterized by inflammatory myopathy and cutaneous skin findings. Cutaneous skin findings include pruritic, red, or violet-colored skin, most commonly found on sun-exposed areas, such as the face, eyelids, elbows, knees, chest, and back.

Evidence of the use of ECP for the treatment of DM is limited to case reports. One case reported that ECP successfully treated an 18-year-old female diagnosed with juvenile DM and was recalcitrant to methotrexate therapy [34,35]. Another case report modifies the ECP procedure by using autologous cryopreserved mononuclear cells in order to reduce apheresis sessions for patients who are treated at distant care facilities. The results of the study proved cryo-ECP to be safe while effectively reducing the number of apheresis sessions [35]. In the ASFA recommendations published in 2016, dermatomyositis was initially labeled in the IV category with a 2C grade of recommendations, meaning “very weak references” [36]. Due to the continuous lack of evidence, the newest ASFA recommendations published in 2019 retired the DM fact sheet from its guidelines.

### 2.4. Eosinophilic Fasciitis

Eosinophilic fasciitis (EF) is a type of rare sclerodermiform syndrome caused by a thickening and inflammation of the muscular fascia and subcutaneous tissue due to eosinophilic infiltrates. Currently, the cause of this disease is unknown, and the current treatment for this disease includes a combination of methotrexate with systemic glucocorticoids [37].

Evidence on the efficacy of ECP for the treatment of EF is limited to case reports (*n* = 6) [38,39,40]. Minciullo et al. administered ECP treatments on two patients who developed eosinophilic fasciitis after undergoing an allogeneic bone marrow transplantation [40]. Both patients reported a significant improvement in their symptoms after 7 and 11 months, with one patient experiencing a normalization of their eosinophil counts [40]. Romano et al. treated three patients with EF that had contraindications or were refractory to first-line treatments [39]. After one year of therapy, two patients experienced a significant clinical improvement in their clinical parameters, while one patient experienced a moderate improvement. All three patients reported an increase in their quality of life and were able to decrease the dose of their immunosuppressants [39]. Partarrieu-Mejias et al. reported a successful ECP treatment for a patient who was diagnosed with steroid-resistant EF [38].

Although the literature shows promising results on the usage of ECP for the treatment of EF, the lack of evidence hinders its recognition by the ASFA and EDF as a safe and effective treatment. Therefore, further studies on this topic are recommended.

### 2.5. Lichen Planus

Lichen planus (LP) is an idiopathic disease that usually affects middle-aged adults, causing inflammation of the skin and mucous membranes. Symptoms are often remembered by the “six P’s”: pruritic, purple, polygonal, planar, papules, and plaques” [41]. The mucous membranes that are commonly affected include oral membranes that are normally characterized by lacy, white, thread-like lesions known as Wickham’s striae. Other presentations of oral lichen planus include plaque-like, papular, erosive, bullous, and ulcerative lesions [42]. The pathogenesis behind this disease has been linked to cytotoxic T-cells that target the skin and mucous membranes, inducing apoptosis of the epithelial basal cells [43]. High-potency topical steroids and retinoic acid are the first-line treatments in treating all forms of this condition, including mucosal, urogenital, and cutaneous erosive lichen planus [44]. However, these therapies have shown to be ineffective against severe subtypes of LP, such as chronic erosive LP.

There are several studies of varying study designs that show promising results that ECP has a positive effect on severe lichen planus, especially in patients suffering from oral erosive lichen planus. The results are summarized in Table 4.

Recent studies include a retrospective study of 11 patients suffering from oral erosive lichen planus that were calcitrant to at least two treatments [54]. Two sessions of ECP were administered for two consecutive days every two weeks at the beginning of the treatment, followed by a tapering off of the treatments, depending on the patient’s therapeutic response and tolerance. The response assessment was measured by complete clinical remission, characterized by the complete healing of erosions and partial remission, and characterized by the regression of functional symptoms but persistence in the same number of erosions that were seen pre-ECP treatment. The results showed that 6 out of 11 patients achieved complete remission at a mean of 5.5 months, and the remaining patients obtained partial remission. Although a relapse was observed in longer time lapses between ECP sessions and after the discontinuation of ECP treatment, the symptoms disappeared once the ECP treatments resumed [54]. The most recent case study validates the earliest case report as well as the two decades of case reports that followed, proving that ECP can be used to treat many severe subtypes of lichen planus [53]. Molochkova et al. reported a patient who was diagnosed with LP pigmentosus that was refractory to topical and intralesional corticosteroids. The patient received four sessions of ECP along with IM chloropyramine and loratadine, and after the fourth treatment, the lesions were noted to have faded, and a decrease in pruritis was also observed [53].

Despite the positive evidence, the ASFA has yet to recognize ECP as an effective treatment for LP. EDF has stated that ECP can be used for oral erosive LP when all conventional therapies, including systemic and topical therapies, have failed. The recommended treatment scheduling includes two successive treatments every 2 weeks for the first 12 weeks, followed by two successive treatments every 4 weeks for an additional 12 weeks. Maintenance dosages should be tailored to the patient and their clinical response. A response assessment is recommended to be based on the disappearance of the lesions.

### 2.6. Lichen Sclerosis

Lichen sclerosis (LS) is a rare, idiopathic, autoimmune pruritic disease that is most commonly seen in pre-pubertal and post-menopausal women [55]. LS is known to cause white, hypopigmented, thin patches on the skin that are most commonly present in the genital area. First-line treatment includes topical steroid ointments.

Evidence of the ECP procedure on LS lesions is limited to three case reports (*n* = 3). ECP proved successful in all three patients with severe refractory LS [56,57,58]. One of these patients had severe extragenital LS that covered the flexor surfaces of the legs, arms, and ankle joints, along with the upper aspects of the arms, legs, and neck [58]. The patient was not only refractory to topical steroids and phototherapy with psoralen + UVA but also presented with severe joint restriction due to LS patches that impaired her ability to walk and plantarflex. A total of 14 ECP cycles were conducted that consisted of two consecutive ECP sessions every 2 weeks for 3 months, followed by one ECP session a month. By the fourteenth cycle, her joint mobility was noted to significantly improve, reaching almost normal joint mobility values. Additionally, the patient was able to walk after the LS patches appeared to remain stable after a 6-month follow-up. The remaining LS cases showed that refractory LS could show remission in as little as four and seven ECP treatments with no adverse side effects [56,57].

Although the results of this case study prove promising, the lack of evidence discourages the ASFA and the EDF from grading the efficacy and usage of this disease in its guidelines. Therefore, more research on the efficacy of ECP on LS should be explored.

### 2.7. Morphea (Localized Scleroderma)

Scleroderma, also known as systemic sclerosis (SS), is an idiopathic autoimmune disease that appears more commonly in women and causes vasculopathy and fibrosis of the dermis. SS can be local or diffuse and may affect visceral organs such as the heart, lungs, and digestive tract. Systemic scleroderma includes diffuse systemic sclerosis and limited cutaneous systemic, which presents with CREST syndromes such as calcinosis and telangiectasis. All forms of systemic scleroderma usually include organ involvement, whereas localized scleroderma, such as morphea and linear scleroderma, are solely confined to the skin. Since the ASFA recommends ECP as a treatment for SS without organ involvement, the review will be focused on the localized scleroderma form of SS. Localized scleroderma (LoS) has an incidence of 3/100,000 individuals each year and can present as linear, round, or oval-shaped plaques that usually appear on the trunk and proximal extremities [59,60]. Although the pathophysiology is complex, it is hypothesized that the inflammation in LoS triggers the connective tissue cells to produce an excessive amount of collagen, leading to fibrosis of the skin [61].

There is mixed evidence regarding the efficacy of ECP on localized scleroderma, which is summarized in Table 5.

Cribier et al. conducted an open clinical trial where seven patients with SS and two patients with severe localized scleroderma received ECP treatments for six months without any simultaneous treatments [62]. A physical assessment after six months showed that the cutaneous manifestations of the SS increased in severity in 4/7 of the patients, with the remaining patients observing no change to their indurations. Visceral involvement did not improve after ECP treatment, and it was concluded that ECP was not effective as a treatment for those with SS. In regards to the localized scleroderma patients, one patient had to terminate their treatment halfway through the study due to the lack of vascular access, and their 6-month assessment was not recorded, while the other patient experienced a decrease in the multitude of their plaques and the visibility of their lesions decreased at 16 months of ECP treatment [62]. In a recent prospective study, twelve patients diagnosed with severe localized scleroderma refractory to treatment received an ECP session every two weeks for six months [65]. The response assessment to treatment included a physical assessment as well as a high-frequency ultrasound session. The results showed that the majority of patients (7/12) saw a decrease in skin thickening/hardening of their plaques, while two patients experienced a halt in their uncontrolled disease progression. One patient did experience a continuation of their localized scleroderma despite treatment, but all patients did not experience any adverse side effects.

Although studies such as Cribier et al. suggest that ECP is ineligible to treat SS, there is an ample amount of evidence that tips the scales in favor of ECP as a treatment for cutaneous manifestations of systemic sclerosis due to its positive effects on the overall improvement or halt in the progression of the skin manifestations of this disease [68,69,70]. Therefore, it has been recognized by the EDF as a second-line treatment for the cutaneous manifestations of SS. However, although the AFSA recognizes the potential benefits of ECP on SS, they require more evidence in order to state that ECP would be an effective second- or first-line treatment for the disease. Additionally, it is yet to be clarified as to whether or not that acknowledgment extends to localized scleroderma, as LoS and SS are found to be two distinct clinical entities that share similar histopathologic and serology findings [71].

### 2.8. Necrobiotic Xanthoma

Necrobiotic xanthoma (NX) is a rare chronic granulomatous disorder that manifests as nodules and yellowish plaques on the skin that are most commonly found in the peri-orbital region [72]. Although the pathophysiology is not well understood, it is associated with monoclonal gammopathy, cholesterol build-up, and hypocomplementemia [73].

There is only one case report to date that explores the possibility of ECP treatment on refractory necrobiotic xanthogranulomas, which reports a positive and beneficial outcome of the use of ECP for refractory NX [74]. However, due to the lack of evidence, NX is not recorded in the EDF or ASFA as a condition that can be treated with ECP. Therefore, further research is suggested to explore the usage of ECP on refractory NX.

### 2.9. Nephrogenic Systemic Fibrosis

Nephrogenic systemic fibrosis (NSF) is a rare idiopathic disease that is commonly found in people who have been diagnosed with advanced kidney failure after exposure to gadolinium, a chemical used in magnetic resonance imaging (MRI). This condition causes thickening and fibrosis of the skin, cutaneous tissues, and sometimes skeletal muscle and can cause swelling, pain, and joint contractures [75].

Evidence for photopheresis as an efficacious treatment for NSF is limited to case series and reports, but all the published literature observed positive effects utilizing ECP (*n* = 16) [76,77,78,79,80,81,82]. One case series reported that all three of the patients treated with ECP found an improvement in their joint mobility and softening of skin indurations in as little as 4 cycles of treatment, with one patient developing complete remission after 16 cycles of therapy [76]. The most recent case study published to date explored the usage of ECP on a NSF patient refractory to sodium thiosulfate, who presented with severe and progressive skin fibrosis that restricted his joint mobility on all four limbs [82]. Therapeutic dosages were utilized according to ASFA’s guidelines for therapeutic dosages for ECP for the treatment of several diseases (two consecutive treatments (one cycle) per week for four weeks). The maintenance therapeutic dosage after a 3-month pause in treatments included one cycle every 2 weeks for 12 weeks and then one cycle monthly. The results show that the patient exhibited a significant improvement in skin lesions and joint mobility after four cycles of ECP. Before ECP therapy, the patient was wheelchair-bound, and after four cycles of therapy, the patient was able to walk approximately 150 ft without any assistance. The patient’s skin lesions and joint mobility were steadily improving until about 14 months of therapy when the benefits seemed to plateau [82].

Due to the sparse amount of studies that prove photopheresis as a beneficial treatment for NSF, the ASFA and EDF recognize ECP as a potential treatment for NSF but under weak evidence, as stated in Table 1.

### 2.10. Pityriasis Rubra Pilaris

Pityriasis rubra pilaris (PRP) is a rare inflammatory and idiopathic papulosquamous disease that is characterized by well-demarcated and distinct orange-hued patches and plaques [83]. These scaly patches are most commonly seen on extensor joint surfaces and extremities that vary in size and severity of scaling. Although the pathogenesis is unknown, several hypotheses include a dysfunction in vitamin A metabolism and inflammatory and/or autoimmune triggers [83].

Only three case reports are noted to date that explore the use of ECP for the treatment of PRP (*n* = 4) [84,85,86]. All of these cases included patients with severe PRP that was refractory to many of the first-line treatments, such as cyclosporin, topical calcineurin inhibitors, corticosteroids, and PUVA therapy. The earliest case report documented a successful treatment of two calcitrant PRP patients, with one patient on a combination of acitretin and ECP and the other on cyclosporin with ECP [86]. Another case report administered one ECP cycle monthly to a patient with refractory ECP and found that after the third ECP session, a physical examination of the skin parameters, such as erythroderma, scaling, itching, and papules, was significantly improved. After the sixth cycle, the application of topical steroids was reduced, and the patient could stop using calcineurin inhibitors. The patient continues to be in partial remission with maintenance with ECP [84]. In contrast, one case report documented a decrease in PRP erythema in the lower limbs but a lack of improvement in her excruciating pruritis when exposed to ECP therapy [85].

There are no guidelines or recommendations to date on the usage of ECP on PRP. Therefore, further exploration is highly encouraged in order to determine the efficacy of ECP in the treatment of PRP.

## 3. Psoriasis

Psoriasis (PS) is a chronic, immune-mediated disease that affects over 60 million people worldwide [87]. PS presents itself in many subtypes on the skin, with the most common cutaneous presentation being plaque psoriasis, characterized as raised skin plaques, mostly on extensor surfaces, the lower back, and the scalp [88]. Although the cause of PS is unknown, psoriasis etiology is found to have a strong genetic component, as it is highly associated with a heritability of 60–90% [89]. Environmental triggers have been observed to induce or exacerbate psoriasis, and T-cells such as TH17 have been found to be upregulated in the onset of psoriasis and disease progression. Current first-line treatments for PS include but are not limited to corticosteroids, calcineurin inhibitors, retinoids, and phototherapy.

Prospective, retrospective, and case reports provided mixed evidence on the ability of ECP to successfully treat cutaneous PS (Table 6).

Wolfe et al. report a de novo development of psoriatic plaques in two patients with cutaneous T-cell lymphoma exposed to interferon-alpha treatment and ECP [95]. However, it has been hypothesized that the incidence is most likely due to the interferon-alpha treatment and not due to ECP. In another prospective study, five patients diagnosed with PS along with psoriatic arthritis were treated with ECP, and all patients showed an improvement in their psoriatic arthritis clinical parameters but no improvement in their skin lesions [92]. In contrast, Vonderheid et al. conducted a prospective trial on four patients with chronic refractory psoriasis who were administered ECP treatments within a range of 6–13 months, and the results showed a partial clearing of skin lesions with an improvement in erythema, induration, and scaling of the lesions [90]. PS flare-ups did still occur when exposed to minor exacerbations after ECP treatment. The immunologic parameters measured a decrease in IL-2 production by peripheral lymphocytes, suggesting that ECP has an anti-inflammatory effect either through the inhibition of IL-2 cytokine production and/or apoptosis of T-cell lymphocytes [90]. The remaining studies in the literature on the treatment of PS with ECP have been positive and in favor of ECP treatment, showing an improvement in the clinical parameters, such as scale, thickness, redness, and magnitude, along with a significant improvement in joint mobility in patients with accompanying psoriatic arthritis [93,97,99].

The ASFA recognizes PS as a grade III, meaning that they recognize the potential benefits of ECP in the treatment of PS; however, external validity and data to support these claims are relatively weak. Similarly, the EDF does not report any recommendations for ECP in the treatment of PS due to “inconclusive evidence”.

### 3.1. Pemphigus Diseases

Pemphigus diseases are rare autoimmune diseases that affect men and women approximately equally. Pemphigus is characterized as flaccid blisters on the skin and mucous membranes due to the immune system attacking the intra-epidermal layer of cells in the stratum spinosum layer of the skin, leading to acantholysis and fluid accumulation [100]. Pathogenesis occurs due to the Th2 and B-cell interaction, leading to the production of IgG auto-antibodies against desmoglein—a desmosomal protein that holds keratinocytes in the epidermis together [101]. The two most common types of pemphigus diseases are pemphigus vulgaris and pemphigus foliaceus. Pemphigus vulgaris (PV) is characterized by blisters on the skin and mucous membranes with auto-antibodies against the desmoglein 1 and/or 3 proteins. In contrast, pemphigus foliaceus (PF) is characterized by blisters on the skin without any mucosal involvement, and only desmoglein 1 is targeted by the immune system [102].

The majority of the ECP studies and case reports on pemphigus disease treatment pertains to PV (*n* = 14) [103,104,105,106,107,108,109,110]. A recent retrospective study on the efficacy of ECP was conducted on eight patients who were diagnosed with drug-resistant PV and who were refractory to first-line treatments, such as corticosteroids and adjuvant therapies, such as colchine [104]. ECP was added to the patient’s systemic therapy and administered for one cycle every 2–4 weeks, and the clinical assessment was recorded every 3 months. The results showed complete remission in all but one of the patients after 2–4 cycles of treatment. All patients were able to decrease their doses of prednisone, with two patients able to completely stop their immunosuppressive therapies. In another retrospective study, drug-resistant PV, as well as PF, was observed to be successfully treated with a monthly cycle of ECP in addition to systemic therapies [103]. All PV patients (*n* = 3) achieved complete remission, while one PF patient achieved partial remission; however, all patients were able to decrease their corticosteroid dosage [103]. Although the majority of the studies validate the hypothesis that ECP is beneficial for the treatment of bullous diseases, one recent case report states that high-intensity ECP failed to treat a patient with refractory PV after 5 months of treatment. Immunophenotyping showed no change in the auto-antibody desmoglein titers, and the patient continued to experience oral lesions that interfered with his oral intake [110].

To date, there are only two case reports and one retrospective study conducted by Wollina et al., mentioned above, that reports on the efficacy of ECP on PF (*n* = 3) [103]. In both cases, a clinical assessment of PF showed blister improvement, and the immunotherapy dosage was able to be lowered after ECP treatment [27,111].

The ASFA has recognized the previous literature and labeled pemphigus vulgaris as a level III for ECP treatment due to the lack of sufficient evidence, and the EDF stated that ECP can be used for PV when it is refractory to conventional systemic therapies. Therapeutic dosing includes one cycle every 2–4 weeks for 12 weeks, then one cycle every 4 weeks. Maintenance intervals include tapering off ECP by adding an additional week before ECP treatment every three months. The response assessment includes measuring auto-antibody titers against desmoglein proteins as well as conducting physical and clinical assessments. To this date, the ASFA and EDF have not recognized ECP as a treatment for PF, which warrants further exploration of the utilization of ECP for the treatment of pemphigus foliaceus.

### 3.2. Autoimmune Sub-Epidermal Bullous Diseases

Sub-epidermal bullous diseases consist of a large group of autoimmune bullae-forming diseases that include bullous pemphigoid and epidermolysis bullosa acquistia. The pathogenesis of sub-epidermal bullous diseases is attributed to auto-antibodies against the hemidesmosomes in the basement membrane, leading to the detachment of the epidermis from the basement membrane zone. This leads to blistering of the skin and mucosal membranes. To date, there is evidence of ECP in the treatment of epidermolysis bullosa acquisita and bullous pemphigoid but no evidence for the remaining sub-epidermal bullous diseases, such as pemphigoid gestationis, mucous membrane pemphigoid, linear IgA bullous dermatosis, and anti-p200 pemphigoid.

#### 3.2.1. Bullous Pemphigoid

Bullous pemphigoid (BP) is the most common sub-epidermal blistering disorder that comprises 80% of the sub-epidermal cases [112]. This disease is often seen in populations of older age and is characterized by tense bullae that can appear anywhere on the skin or mucosal membranes [112]. Patients diagnosed with this condition also experience pruritis before the onset of the bullae.

There are only two studies that report the use of ECP for the treatment of BP (*n* = 5) [103,113]. In addition to exploring the usage of ECP on PV and PF, Wollina et al. also administered ECP therapy to three patients with refractory BP. The results showed complete remission after 1–4 cycles, and immunosuppressive therapy was able to be lowered with no adverse effects. Tripodi et al. reported that a pediatric case of refractory BP achieved complete and long-term remission with ECP, administered alongside plasma exchange and corticosteroid therapy [113]. Immunosuppressive therapy was also able to be tapered off at an 18-month follow-up.

Due to the lack of evidence, the ASFA and EDF have not recognized BP as a condition that has the potential to be effectively treated with photopheresis.

#### 3.2.2. Epidermolysis Bullosa Acquisita

Epidermolysis bullosa acquisita (EBA) is a chronic blistering disease that affects 20,000–50,000 individuals worldwide [114]. This autoimmune disease is characterized by auto-antibodies that target type VII collagen in the dermal–epidermal junction, leading to detachment of the epidermis. Symptoms of EBA include skin fragility, blisters, erosions, and scarring. The two most common types of EBA are classic mechanobullous, characterized by bullae and erosions at the affected site, and the inflammatory form, which presents as an inflammatory bullous eruption that looks similar to that of other sub-epidermal blistering diseases [115].

There are few prospective studies, retrospective studies, and case reports pertaining to ECP treatment for EBA. These publications have confirmed ECP’s efficacy as a treatment for refractory and severe cases of this disease (*n* = 13) [104,116,117,118,119,120]. Camara et al. documented a case of severe EPA with severe refractory EBA with ocular involvement that was compromising the patient’s vision [119]. After 32 treatments of ECP, all of the patient’s EBA symptoms disappeared, with the exception of his cicatricial alopecia, and the patient was able to cease all concomitant treatments. Additionally, the most recent ECP publication for EPA involved a 12-year-old girl whose integumentary symptoms were treated after several rounds of ECP treatment, suggesting the possibility that ECP can be used on pediatric patients to treat dermatologic diseases [117].

Due to the weak evidence that supports ECP treatment for this condition, the ASFA does not recognize this disease in their guidelines. In contrast, the EDF states that ECP can be used as an alternative treatment when other front-line treatments fail. The recommended therapeutic regimen includes one cycle every 2–4 weeks for 12 weeks, then one cycle every 4 weeks, with tapering maintenance therapy of additional 1-week intervals after every three months. An assessment of the treatment response includes clinical and photographic assessments.

### 3.3. Scleromyxedema

Scleromyxedema (SX), also known as Arndt–Gottron’s disease, is a rare idiopathic chronic cutaneous disease that results in abnormal accumulation of mucin in the skin and affects adults 30–70 years of age, without any predilection for specific gender or race [121]. Scleromyxedema is characterized by the eruption of symmetrical 2–3 mm waxy firm papules that have histological evidence of mucin deposition, fibroblast proliferation, as well as fibrosis [122]. Although the pathophysiology is unknown, researchers have identified IgG paraprotein in SX patients’ serology, although there has not been any relationship proven to be associated with decreased IgG paraprotein levels nor improvement in clinical manifestations [123]. First-line therapies include IV immunoglobulin administration (IVIG) and systemic glucocorticoids [124].

The majority of studies on the potential use of ECP on SX have been explored through case reports and a retrospective study (*n* = 7). All of the studies reported that SX was successfully treated using ECP [106,125,126,127,128,129]. Some case reports even observed an almost complete remission of SX after 6–12 months of ECP therapy [125,126,127]. Additionally, serologic evaluations also showed a significant drop to minimal or no IgG paraprotein levels [128,129]. Although these publications encourage the use of ECP to treat SX, Durani et al. reported that one of the SX patients relapsed after 4 weeks of skin improvement with ECP monotherapy [126]. Oral cyclophosphamide was added to his treatment regimen, and his skin continued to improve, suggesting ECP should be used in combination therapy.

Like many of the other dermatologic diseases, there is not enough evidence for the ASFA and EDF to recognize ECP as a safe and efficacious treatment for SX. The EDF recognizes that studies on scleromyxedema have been conducted but states there is inconclusive evidence.

### 3.4. Solar Urticaria

Solar urticaria is a very rare chronic allergic reaction to sun exposure that causes pruritis, wheals, and erythema in sun-exposed areas. The cause of solar urticaria is not clearly defined, but the pathogenesis is stated as a type 1 IgE-mediated hypersensitivity reaction that involves Th2 cells interacting with B-cells, causing an inflammatory response and mast-cell activation [130]. First-line therapy includes antihistamines and corticosteroids.

To this date, there is only one case report on the treatment of solar urticaria utilizing ECP [131]. This case report talks about a patient with severe solar urticaria who was extremely sensitive to both UVA radiation and visible light, with first-line treatments such as oral antihistamines and oral cyclosporin proving ineffective. After nine treatment cycles, the patient’s minimal urticaria dose increased from 7 J/cm^2^ to 22 J/cm^2^ UVA. The clinical assessment included a significant improvement in the multitude and magnitude of wheals and associated symptoms, such as pain, pruritis, and fatigue.

Since there is only one study that discusses the usage of ECP on solar urticaria, the ASFA and EDF have not recognized photopheresis as a potential treatment for this disease. Therefore, more studies should be conducted in order to further increase our knowledge of the efficacy of ECP on solar urticaria.

## 4. Photopheresis Efficacy, Tolerability, and Cost Effectiveness

Although the preliminary evidence and case reports from the aforementioned publications state ECP can be a safe and efficacious treatment, there are not enough clinical trials to validate these findings for a majority of these dermatologic diseases. Additionally, the variation in ECP regimens in pilot studies to treat the same dermatologic disease makes it difficult to replicate and compare the clinical outcomes between studies.

ECP has been proven to be an efficacious treatment in autoimmune diseases that allows patients to decrease and even discontinue any concomitant immunosuppressive therapy, which can ultimately result in a decrease in morbidity and infection, leading to increased overall survival. Photopheresis can also lower the risk of infection due to T-cell retention of its antigen-specific responses, allowing ECP T-cells to continue to amount an immune response to vaccinations and other antigenic presentations [132,133].

Photopheresis has been observed as a safe and tolerable treatment, with relatively limited to no adverse side effects reported in the literature. The EDF also reported minimal side effects in its ECP guidelines, but some patients did report side effects such as hypotension, tachycardia, low-grade anemia, and thrombocytopenia [134]. Additionally, a recent publication measured the effects of ECP on vitamin D levels and found that a vitamin D deficiency could result after long-term usage of ECP [135]. Therefore, vitamin D levels are advised to be monitored during ECP treatment. Contradictions for ECP therapy include pregnancy, sensitivity to psoralen compounds, photosensitivity, aphakia, and a history of heparin-induced thrombocytopenia [134].

The potential drawbacks to using ECP for therapy include scheduling and geographical location. Some patients may find it difficult to come into a care center at frequent and regular intervals to receive treatment, especially if they live far away or have to make a long commute. Recent advancements to potentially address this lack of accessibility include the study of autologous cryopreserved mononuclear cells for ECP treatments [35,136]. This will allow most of the treatment to be conducted in a care setting that is in close proximity to one’s residence, resulting in a reduction in the number of photopheresis sessions conducted at the primary care center. Although this allows photopheresis to be more easily accessible, patients still run into the problem of ECP being too expensive. The average cost for ECP is USD 3045.61 per treatment, and therefore, patients have run into problems with insurance companies refusing to pay for these treatments [83,137].

## 5. Conclusions

This is the first review to our knowledge that provides a comprehensive analysis of all the dermatologic diseases that have been treated with ECP. Heidrum et al. conducted a brief review of the miscellaneous indications for ECP; however, it did not provide the in-depth analysis that we aimed to accomplish in this review. ECP has been recognized by the APSA and EDF as a safe and efficacious treatment for a variety of dermatologic and autoimmune diseases. Further evidence is needed in order to validate the current findings of ECP efficacy for the remaining dermatologic diseases that lack sufficient evidence for ECP approval. Further research could explore photopheresis in the treatment of other autoimmune dermatologic diseases, such as the remaining sub-epidermal bullous diseases and vitiligo, as psoralens such as MOP-8 have been hypothesized to stimulate melanogenesis in these patients [138].

## Figures and Tables

**Table 1:** Guidelines for using ECP in dermatologic diseases.

Disease	ASFA 2019 ECP Guidelines	EDF ECP Guidelines 2020		
		Indication	Initial Treatment Schedule	Response Assessment
Atopic Dermatitis (AD)	III (2A)	Second-line and if >18 months’ duration; SCORAD > 45; severe refractory AD	1 cycle every 2 weeks for 12 weeks	SCORAD assessment every other week for 12 weeks, then once a month or longer
Cutaneous Lupus Erythematosus	No recommendations	No recommendations, but “preliminary results represent ECP as an innovative, effective, and safe therapeutic option for treatment of LE”		
Cutaneous T-Cell Lymphoma	Erythrodermic: I (1B) Non-erythrodermic: III (2C)	First-line treatment for erythrodermic stage IIIA or IIIB	1 cycle every 2 weeks, then every 3–4 weeks for 6–12 months	Lab measurements and visual assessment every 3 months. Treatment should be initiated for at least 6 months to be determined ineffective
Epidermolysis bullosa acquista	No recommendations	Refractory with conventional systemic therapies	1 cycle every 2–4 weeks for 12 weeks, then 1 cycle every 4 weeks	Validated scoring systems and visual/photographic assessment
Lichen Planus	No recommendations	Consider for refractory oral erosive lichen planus	1 cycle every 2 weeks for 0–12 weeks, then 2 consecutive treatments every 4 weeks for 12–24 weeks	Disappearance of oral lesions
Scleroderma (Systemic Sclerosis)	III (2A)	Second-line, combination therapy or mono-adjuvant therapy; recommended for skin treatment without organ involvement	1 cycle every 4 weeks for 12 months	Validated scoring systems and visual/photographic assessment
Nephrogenic Systemic Fibrosis	III (2C)	“inconclusive evidence”		
Pemphigus Vulgaris	III (2C)	Refractory with conventional systemic therapies	1 cycle every 2–4 weeks for 12 weeks, then 1 cycle every 4 weeks	Auto-antibody titers and validated scoring systems and visual/photographic assessment
Pityriasis Rubra Pilaris	No recommendations	“ECP has shown to be effective”		
Psoriasis	III (2B)	“Inconclusive evidence”		
Scleromyxoedema	No recommendations	“Inconclusive evidence”		

1 cycle = 2 consecutive days; SCORAD = “SCORing Atopic Dermatitis” tool for assessing severity of AD.

**Table 2:** Summary of ECP treatment for atopic dermatitis.

Author/Year	Study Design	Number of Patients (*n*)	Initial Clinical Symptoms	Initial ECP Treatment	Treatment Response
Prinz et al. [11] 1994	Case Series	3	All patients had life-long history of AD that became refractory to first-line treatments *	1 cycle every 4 weeks, then after 6 cycles, then 1 cycle every 6 weeks.	- 2/3 CR * by 5th cycle - 1/3 PR * - Decrease in cutaneous inflammatory activity - Increase in IgE - IgG, IgM, IgA, and circulating lymphocyte profile unchanged
Richter et al. [12] 1998	Case Series	3	All patients had severe AD, recalcitrant to first-line treatments *	1 cycle every 2 weeks for 5 cycles	- PR * in all patients - Decrease in eosinophil cationic protein and IgE
Mohla et al. [13] 1999	Case Report	1	Life-long AD with severe manifestations over past 20 years diminishing QoL *. Resistant to first-line treatments *	1 cycle every 2 weeks for 5 weeks, then every 4 weeks until week 16.	- CR * - Significant improvement in QoL *
Radenhausen et al. [14] 2003	Case Series (retrospective)	10	All patients had severe and refractory AD with a SCORAD * (87.3 ± 9.1)	1 cycle every 2 weeks with oral MOP for 5 cycles	- Significant decrease in SCORAD * (87.3 ± 9.1)
Radenhausen et al. [15] 2003	Bicentre, clinical trial	35	All patients had severe AD (SCORAD * 74.4 ± 15.5) resistant to first-line therapy *	1 cycle every 2 weeks for 6–10 cycles	- Significant decrease in SCORAD * (36.8 ± 16.8, *p* < 0.05) - 24/33 patients saw clinical improvement - Decrease in eosinophil cationic protein (27%) - Patients with no clinical response measured high IgE levels before and during therapy
Sand et al. [16] 2007	Single arm, open-label trial	7	All patients had severe AD, SCORAD * > 45 (77.7 ± 8.5) for at least a year that was refractory to first-line and second-line therapies within that year	1 cycle every 2 weeks for 12–20 weeks	- Significant decrease in SCORAD * (55.6 ± 10.3) - FACT score showed significant improvement in QoL * (64.8 to 72.9, *p* < 0.05) - Patients report clinical improvement in skin conditions
Hjuler et al. [17] 2010	Case series (retrospective)	6	All patients had severe recalcitrant AD	1 cycle every 4–8 weeks for approximately 6 years	- All patients saw clinical improvement - One patient reported CR *
Wolf et al. [18] 2013	Prospective trial	10	All patients had disease duration of at least 1 year, SCORAD * > 45 (64.8 ± 18.9), and resistance to first-line therapies *	1 cycle every 2 weeks for 12 weeks, then every 4 weeks until week 20.	- Decrease in SCORAD * (54.5 ± 22.8) - No statistical significance in QoL * instruments, including FACT * score
Rubegni et al. [19] 2013	Case Series (retrospective)	7	All patients were refractory to first-line treatments for over 6 months	1 cycle every 2 weeks for 3 months	- Decrease in SCORAD * from baseline with long-lasting stabilization in (4/7) patients.
Chiricozzi et al. [20] 2014	Case Series	3	All patients characterized with severe AD (SCORAD * 50.3 ± 8.6) and (Pruritic VAS * 73.3 ± 11.5) recalcitrant to first- and second-line therapies	Cycle varied between patients; 4, 10, and 20 cycles between 3–12 months	- Decrease in SCORAD * (24 ± 8.0). - Decrease in pruritic VAS * (43.3 ± 15.28)
Koppelhus et al. [21] 2014	Randomized cross-over study	20	All patients had severe AD (SCORAD * 69 ± 16) refractory to first-line and second-line treatments with a pruritis score of (6.5 ± 1.8)		- Decrease in SCORAD * (37 ± 16, *p* = 0.4) - Decrease in pruritis (2.4 ± 1.8, *p* = 0.6) - 6% mean reduction in IgE levels - 9% reduction in eosinophilic granulocytes - 67% responded positively to ECP
Meyersburg et al. [22] 2019	Case Series	2	All patients had severe AD with a SCORAD * > 50	First patient: 1 ECP treatment for first 8 weeks, then 15 cycles every 2 weeks. Second patient: 1 cycle bi-monthly, then one cycle monthly for 6 cycles	- Both patients experienced a 41% and 21% decrease in SCORAD *.
Gambichler et al. [23] 2022	Retrospective single-center chart review	60	Severe AD	Patients had a median number of 14 ECP cycles (range 4–23) within a maximum 1 year of treatment	- Clinical improvement in majority of patients - Leukocytes and lymphocytes were found to remain decreased after one year of ECP treatment (*p* = 0.014, *p* = 0.0012) - A significant decrease in eosinophils and eosinophil cationic protein levels (*p* = 0.011, *p* = 0.0017) - IgE and lactate dehydrogenase levels significantly decreased (*p* < 0.00001 and *p* = 0.00007)
Summary		*n* = 167		Variable cycle schedule	

* CR = complete remission; PR = partial remission; SCORAD = “SCORing Atopic Dermatitis” tool for assessing severity of AD; first-line therapies include but not limited to topical steroids, topical calcineurin inhibitors, and phototherapy (UVA, UVB, and PUVA); second-line therapies include but not limited to systemic steroids or cyclosporine; QoL = quality of life; FACT = Functional Assessment of Chronic Illness Therapy Survey; Pruritic VAS = Pruritus Visual Analog Scale.

**Table 3:** Summary of ECP treatment for cutaneous lupus erythematosus.

Author/Year	Study Design	Number of Patients (*n*)	Initial Clinical Symptoms	Initial ECP Treatment	Treatment Response
Knobler et al. [25] 199	Pilot study	8	Patients with cutaneous LE, along with other SLE symptoms such as arthritis and myalgias	1 cycle monthly for 6 months	- 7/8 patients saw significant positive response to treatment - Auto-antibodies and lab parameters were unchanged - Clinical activity score decreased from median of 7 to median of 1 (*p* < 0.05)
Knobler et al. [26] 1994	Clinical trial	10	All patients with SLE that was not life-threatening in the short run and had mild to moderate disease activity with flare-ups that occurred with attempted reduction of first-line treatments *	1 cycle every 4 weeks for 6 months followed by 1 cycle every 8 weeks for 6 months	- 8/10 patients completed the study (1 dropped out for personal reasons; another patient passed away during the study) - 7/8 patients saw significant improvement in skin manifestations in 4–6 months - Patients were able to decrease their dose of immunosuppressants and steroids - No notable changes in serology measurements during or after this trial
Licht et al. [27] 1996	Case report	1	Patient diagnosed with SLE and urticarial vasculitis with severe side effects under immunosuppressive therapy with azathioprine and prednisone	ECP with concomitant immunomodulatory therapy	- Clinical picture improved, and immunosuppressive drugs were able to reduce
Richter et al. [28] 1998	Case report	1	Patient with discoid LE that did not respond to conventional therapy	14 cycles at 4-week intervals	- CR * on face, chest, and back with a cease in hair loss
Wollina et al. [29] 1999	Case report	2	First patient diagnosed with subacute cutaneous LE, and second patient diagnosed with chronic discoid LE. Both refractory to conventional therapies	1 cycle bi-monthly with oral MOP	- CR * with prolonged remission of 18 and 11 months.
Richard et al. [30] 2002	Case report	1	Patient diagnosed with subacute lupus without systemic disease that was refractory to first-line therapies. Patient presented with erythematous and squamous patches on face and neckline with hyperpigmented lesions on arms and shoulders	ECP treatments initiated up to nine months	- CR * attained after two months; lab abnormalities remained unchanged - Relapsed cutaneous lesions occurred at nine months, and treatment was discontinued
Boeckler et al. [31] 2009	Case report	1	Female patient was diagnosed with subacute discoid cutaneous LE	1 cycle every 15 days	- CR * after 4 cycles - Prolonged remission up to 18 months. - Marked changes in levels in lab parameters and auto-antibodies
Morruzzi et al. [32] 2009	Case series	4	All patients diagnosed with refractory cutaneous lupus (1 subacute LE and 3 chronic LE)	1 cycle every 2 weeks	- CR * in two patients and PR * in two patients after 2–3 months of treatment - All concomitant treatments able to be stopped
Summary		*n* = 28		Variable cycle scheduling	

* First-line treatments include nonsteroidal anti-inflammatory drugs, lose-dose steroids, oral cyclophosphamide, chloroquine, and oral azathioprine; CR = complete response; PR = partial response.

**Table 4:** Summary of ECP treatment for lichen planus.

Study	Study Design	Number of Patients (*n*)	Initial Clinical Symptoms	Initial ECP Treatment	Treatment Response
Becherel et al. [45] 1998	Open prospective study	7	Oral erosive lichen planus	2 treatments weekly for 3 weeks, then tapered according to patient’s needs	- All patients had CR * in a mean of 12 months - Hemoglobin and platelets remained unchanged, B lymphocytes and NK cells remained unchanged, with a decrease in lymphocytes
Bussel et al. [46] 2001	Open prospective study	10	Oral erosive lichen planus with half of the patients presenting with vulval erosions	1 cycle weekly for 3 weeks, then tapered according to patient’s needs	- Patients saw clinical improvement with an improvement in functional signs in a mean number of 14 sessions. - CR * in 9/10 patients and PR * in the remaining patient - Hemoglobin and platelets remained unchanged, B lymphocytes and NK cells remained unchanged, with a decrease in lymphocytes
Kunte et al. [47] 2005	Case reports	4	All patients had erosive oral lichen planus that was resistant to treatment	1 cycle every 2 weeks	- All patients saw improved mucosal lesions and clinical symptoms after 7–9 cycles - Lesions temporarily worsened in 2 patients following dental procedures - One patient remained in CR after 19 cycles and remained in remission for 9 months
Guyot et al. [48] 2007	Case series	12	All patients had erosive oral lichen planus recalcitrant to conventional immunosuppressive therapies	1 cycle every 3 weeks, then treatment tapered according to individual	- CR * in 9/12 patients - PR * in 3/12 patients - Long-term follow-up over three years found lesion recurrences when ECP became less frequent or stopped
Marchesseau- Merlin et al. [49] 2008	Case reports	2	One patient diagnosed with erosive oral lichen planus for four years. Second patient presented with cortico-dependent oral and genital erosive lichen planus with cutaneous lesions	Total of 9 and 20 ECP sessions, respectfully	- One patient experienced subjective improvement and oral lesions were stabilized - Second patient experienced CR * without the use of corticotherapy
Zingoni et al. [50] 2010	Case report	1	Patient was diagnosed with multi-resistant and painful erosive LP on oral and genital mucosa	1 cycle every 3 weeks	- CR * was achieved in 8 months with substantial re-epithelization of vulvar erosions
Elewa et al. [51] 2011	Case report	1	Patient was diagnosed with disseminated lichenified papules on mouth and genital mucous membranes with cicatricial alopecia of the scalp	1 cycle every week for 6 cycles	- Patient saw a clinical improvement in lichenified papules
Serikova et al. [52] 2018	Randomized control trial	40	Patients with severe forms of oral lichen planus that were erosive-ulcerative and exudative-hyperemic	ECP daily for 10 treatments	- 19 patients experienced a reduction or disappearance in pain and reduced inflammation after 30 days
Molochkova et al. [53] 2019	Case report	1	Female patient diagnosed with LP pigmentosa, refractory to topical and intralesional corticosteroids	4 sessions of ECP administered on alternating days	- Patient experienced a notable decrease in pruritis and faded lesions after the fourth treatment
Birckel et al. [54] 2020	Retrospective study	11	All patients suffer from oral erosive lichen planus that are recalcitrant to at least two treatments.	1 cycle every 2 weeks, then tapering off of treatments depending on patient’s therapeutic response and tolerance	- CR * achieved in 6/11 patients at a mean of 5.5 months - Remaining patients achieved PR - Relapse observed in longer time lapses between ECP sessions and after discontinuation of ECP treatment; symptoms disappeared once ECP treatments resumed
Summary		*n* = 89		Variable cycle scheduling	

* CR = complete response; PR = partial response.

**Table 5:** Summary of ECP treatment for localized scleroderma.

Study	Study Design	Number of Patients (*n*)	Initial Clinical Symptoms	Initial ECP Treatment	Treatment Response
Cribier et al. [62] 1995	Open clinical trial	9	Seven patients were diagnosed with SS, and two patients with severe morphea	1 cycle every 2–4 weeks for 6 months	- Cutaneous manifestations of the SS increased in severity in 4/7 patients, and no visceral improvement observed - Unchanged lesions were observed in the remaining SS patients - One LS patient had to terminate treatment halfway through the study due to the lack of vascular access - Other LS patient experienced a decrease in multitude and visibility of their plaques at 16 months of ECP treatment
Schlaak et al. [63] 2008	Case report	1	Severe, resistant morphea involving trunk and extremities, progressing to sclerotic plaques and scars with bullous eruptions	1 cycle every 2 weeks for 6 cycles, then tapered off to longer intervals	- CR * of erosions after 6 months of treatment with alleviation of pain
Neustadter et al. [64] 2009	Case report	1	Female diagnosed with generalized, refractory deep morphea, interfering with ADLs *; waking patient in the night due to pain and discomfort	1 cycle every 2 weeks, then tapered off to longer intervals	- After 2 months, patient reported increased mobility and energy levels - Plaques on abdomen resolved with clearance of hardened lesions on upper and lower extremities.
Merlin et al. [35] 2011	Case report	3	One patient was diagnosed with juvenile localized scleroderma	Cryopreserved ECP was utilized to decrease the number of apheresis sessions	- Cryo-ECP was safe, feasible, and effective at maintaining efficacy of regular ECP while decreasing the number of apheresis sessions
Just et al. [65] 2013	Prospective single-center clinical study	12	All patients diagnosed with severe refractory localized scleroderma	1 cycle every 2 weeks for 6 months	- Majority of patients (7/12) saw a decrease in skin thickening/hardening of their plaques - Two patients experienced a halt in their uncontrolled disease progression - One patient experienced a continuation of their LoS despite treatment
Pileri et al. [66] 2014	Case report	1	Female patient presented with indurated, erythematous plaques on lower and upper extremities for 4 months, refractory to methotrexate and steroids	1 cycle every 2–8 weeks for 16 months	- CR * - ECP stopped, and CR * was prolonged after 1-year follow-up
Papp et al. [67] 2016	Prospective study	25	Nine patients diagnosed with diffuse cutaneous SS * for a mean of 1.9 years. Sixteen healthy patients served as the control group for laboratory results	1 cycle every 6 weeks for 1 year	- Improvement in skin score after 6 months - Increase in Treg cells, CD4 + CD25 and Tr1 observed up to one year; values then plateaued - % of Th17 cells decreased
Summary		*n* = 52		Most patients received 1 cycle every 2 weeks, ranging from 6 months to 16 months	

* SS = systemic sclerosis; ADLs = activities of daily living; CR = complete remission.

**Table 6:** Summary of ECP treatment for psoriasis.

Study	Study Design	Number of Patients (*n*)	Initial Clinical Symptoms	Initial ECP Treatment	Treatment Response
Vonderheid et al. [90] 1989	Case series	4	All patients experienced chronic refractory psoriasis vulgaris without arthropathy	Oral MOP with treatment duration ranging from 6–13 months	- PR * of skin lesions observed - % of lesion involvement was 40–80% of baseline scores post-ECP - PS flare-ups occurred when exposed to minor exacerbations after ECP treatment - Decrease in IL-2 production by peripheral lymphocytes, suggesting anti-inflammatory effect, either through inhibition of IL-2 cytokine production and/or apoptosis of T-cell lymphocytes
Vonderheid et al. [91] 1990	Case Series	4	All patients had chronic refractory plaque-type psoriasis without arthropathy	1 cycle of ECP biweekly for 6 to 13 months Methotrexate concurrently administered up to 6 months	- Two patients’ lesions improved 23% and 62% of baseline values; concurrent methotrexate treatment had to be maintained due to relapse of lesions with ECP alone - Remaining patients experienced improvement in body surfaces by 50% at 4 months of treatment; they experienced relapse due to reasons unrelated to treatment - Prolonged ECP treatment led to decreased production of IL-2 and skin reactivity to recall antigens
Wilfert et al. [92] 1990	Case Series	5	All patients diagnosed with long-standing sero-negative arthritis and psoriasis of the skin resistant to conventional therapy		- Marked reduction in viability, proliferation, and mitogen response - Slight to moderate clinical improvement in four of five patients with arthralgia features. - Skin lesions did not respond to photopheresis
Misa et al. [93] 1992	Case Report	1	Male patient has a 13-year history of psoriasis and PA with refractory to first- and second-line therapies	1 cycle every 4 weeks for 1 year	- 6 months of treatment showed moderate improvement in both skin lesions and arthropathy - Oral MTX dose was able to be reduced
Misa et al. [94] 1994	Case Reports	2	One PS patient report was summarized in Misa et al. (1992) [93] The second patient was diagnosed with cutaneous psoriasis that preceded PA * by 9 years. Third patient had palmoplantar PS that preceded PA by 7 years. All patients refractory first-line treatments	1 cycle every 4 weeks for 1 year	- Second patient: ESR normalization and PASI * improved after 1 year of treatment; methotrexate dose was reduced - Third patient: Initial improvement after 6 procedures, but relapse occurred at 8 months, requiring increasing methotrexate dose; 1 year of treatment showed no difference in lab values or lesions from baseline
Wolfe et al. [95] 1995	Case Report	2	Both patients were diagnosed with cutaneous T-cell lymphoma and exposed to interferon-alpha treatment and ECP	Only the first patient’s ECP regimens was reported, and patient received monthly photopheresis	- Both patients developed de novo psoriatic plaques after treatment with ECP - Hypothesized that incidences are most likely due to interferon-alpha treatment and not due to ECP
Vahlquist et al. [96] 1996	Prospective study	8	All patients were diagnosed with psoriasis and sero-negative arthritis	1 cycle on weeks 2, 4, 8, and 12, followed by monthly ECP and PUVA for 12 weeks; oral MOP	- PASI * index significantly decreased after 24 weeks (6.5 ± 1.8 vs. 5 ± 5, *p* < 0.05) - Half of the patients experienced clinical improvement in joint symptoms that lasted for a least 12 months
Molochkov et al. [97] 2012	Comparative Study	93	All patients were diagnosed with PS-associated PA * 52 patients assigned to study group treated with ECP; 41 patients randomly assigned in the control group	Four ECP sessions administered on alternating days to study group along with pharmacotherapy Control group received pharmacotherapy alone	- 49/52 patients of study group experienced improvement in PS - Decreased mean PASI score in study group (19.7 ± 3.4 to 6.7 ± 2.1, *p* < 0.05) - Control group experienced an improvement in PASI * score to a lesser degree (19.2 ± 3.7 to 12.2 ± 3.1, *p* < 0.05)
Demiriz et al. [98] 2013	Case Report	1	Patient diagnosed with acute GVHD presented with psoriasis vulgaris lesions refractory to cyclosporine and methylprednisolone Histology showed features of both psoriasis and acute GVHD	1 cycle every 2 weeks for 2 months, then 1 cycle monthly combined with cyclosporine and methylprednisolone	- CR after 2 months of treatment - Continuous remission after 18 months of treatment
Esme et al. [99] 2021	Case Report	1	Patient was diagnosed with non-Hodgkin’s lymphoma and PS that was refractory to first-line therapies	4 ECP sessions within the first month, then 1 cycle every 2 weeks in the second month for a total of 12 ECP treatments	- CR * with a decreased PSAI score (19 vs. 6) and DLQI score (17.5 vs. 2.8) after 2.5 months
Summary		*n* = 121		Variable ECP cycle between studies	

* PA = psoriatic arthritis; PASI = Psoriasis Area Severity Index; CR = complete remission; first-line therapies include methotrexate, narrowband ultraviolet B, acitretin, and cyclosporine.

## Data Availability

No new data were created in this study.

## Supplementary Materials

The following supporting information can be downloaded at: https://www.mdpi.com/article/10.3390/ijms25053011/s1.
