# Supplementary material for: Extracorporeal Photopheresis in Dermatological Diseases
Source: Int J Mol Sci. 2024 Mar 5;25(5):3011. doi: 10.3390/ijms25053011 (PMC10932455; doi:10.3390/ijms25053011)
Supplement: Supplementary file 1 [file ijms-25-03011-s001.zip › ijms-2874330-supplementary.pdf]

Supplementary Files for Extracorporeal Photopheresis in Dermatological Diseases

Supplementary Table S1. APSA Guideline for Therapy Staging.

Category	Definition
**I**	Considered as **first-line** treatment; used as monotherapy or concomitant treatment
**II**	Considered a **second-line** treatment; used as monotherapy or concomitant treatment
**III**	Literature shows beneficial treatment but insufficient evidence. Treatment decision is based on individual scenario.
**IV**	Literature states ECP is harmful or ineffective. IRB approval is needed in order to administer treatment

Supplementary Table S2. APSA Guideline for Strength and Quality of Evidence.

Grading Recommendation	Quality	Implementation
**Grade 1A**	Strong recommendation with high quality evidence	Can apply to most patients without concern
**Grade 1B**	Strong recommendation with intermediate quality evidence	Can apply to most patients without concern
**Grade 1C**	Weak recommendation with high quality evidence	Strongly recommended but recommendation can change as more evidence becomes available
**Grade 2A**	Weak recommendation with high quality evidence	Application dependent on circumstance and patient’s preference
**Grade 2B**	Weak recommendation with intermediate-quality evidence	Application dependent on case-by-case basis and patient’s preference
**Grade 2C**	Weak recommendation with low-quality or very low-quality	Other alternatives should be considered
